# Efficiency of non-ionic surfactants - EDTA for treating TPH and heavy metals from contaminated soil

**DOI:** 10.1186/2052-336X-11-41

**Published:** 2013-12-20

**Authors:** Mansour Baziar, Mohammad Reza Mehrasebi, Ali Assadi, Mehran Mohammadian Fazli, Mohammad Maroosi, Fooad Rahimi

**Affiliations:** 1 Department of Environmental Health Engineering, School of Public Health, Tehran University of Medical Science, Tehran, Iran; 2 Department of Environmental Health, Faculty of Health, Zanjan University of Medical Sciences, Zanjan, Iran; 3 Department of Health, Neyshabur University of Medical Sciences, Neyshabur, Iran

**Keywords:** Soil, Non-ionic surfactants, Chelating agent, Total petroleum hydrocarbons, Heavy metals

## Abstract

Introduction of fuel hydrocarbons and inorganic compounds (heavy metals) into the soil, resulting in a change of the soil quality, which is likely to affect use of the soil or endangering public health and ground water. This study aimed to determine a series of parameters to remediation of TPH and heavy metals contaminated soil by non-ionic surfactants- chelating agents washing process. In this experimental study, the effects of soil washing time, agitation speed, concentration of surfactant, chelating agent and pH on the removal efficiency were studied. The results showed that TPH removal by nonionic surfactants (Tween 80, Brij 35) in optimal condition were 70–80% and 60–65%, respectively. Addition of chelating agent (EDTA) significantly increases Cd and Pb removal. The washing of soil by non- ionic surfactants and EDTA was effective in remediation of TPH and heavy metals from contaminated soil, thus it can be recommended for remediation of contaminated soil.

## Introduction

Soil pollution by fuel hydrocarbons and inorganic compounds are major types of pollution [1-3]. Total Petroleum Hydrocarbons (TPH) is a big class of fuel hydrocarbons that originally come from crude oil and are found in large levels in diesel fuels. Some of these compounds in exposure with human and animals can cause cancer, disorder central nervous system and also have harmful effects on liver and lungs [4]. The most common sources of TPH in the environment are accidental releases of crude oil and its products, petroleum refining wastes, petroleum refining products and leaching of oil storage tanks [4-7]. The presences of inorganic compounds such as heavy metals especially lead and cadmium in soil can pose a significant threat to human health and ecological systems [8]. Cadmium and lead are commonly encountered hazardous heavy metals and are in the EPA’s list of priority pollutants [9,10]. Heavy metals are relatively motionless and persistent in soils as a result of precipitation or adsorption reactions. Industrial facilities are sources of introducing heavy metals into the soil. There are many soil treatments technical methods for contaminated soils including bioremediation, soil washing, soil flushing, thermal desorption, thermal destruction and vapor extraction [11,12]. Soil washing is a simple and effective technology for rapid removal of hydrocarbons and heavy metals adsorbed into soil [13]; hence it has been successfully practiced for many years [14]. Literature showed that the soil washing by surfactant can be high effective for hydrophobic pollutants and heavy metals [15]. Metals, semi-volatile organics, PAHs, pesticides and PCBs can be treated by soil washing technique [16,17]. Surfactants are amphiphilic molecules with a hydrophilic head group and a hydrophobic tail group. They can be: anionic, non-ionic, cationic and amphoteric [18,19]. The major reasons of using non-ionic surfactants (Tween 80 & Brij 35) in this study include; biodegradable properties, cost-effective and low tendency to flocculants clay particles in soil compared to ionic surfactants. These surfactants enhance the solubility of hydrophobic organic compounds by partitioning them into the hydrophobic cores of surfactant micelles [20-22]. Chelating agents like EDTA and NaCl sometimes are entered to soil washing due to high efficiency of metal extraction, high thermodynamic stabilities of the metal complexes formed, good solubility of metal complexes and normally low adsorption of the chelating agents and their metals complexes on soils [8].

This process can be affected by several factors including agitation speed, washing time, surfactant concentration, and liquid - soil ratio [23]. The main objective of this study was investigation of non-ionic surfactants and chelating agents on removal of TPH (C_10_ – C_28_) and heavy metals (Pb and Cd) in contaminated soil.

## Materials and methods

### Soil preparation and experimental design

The sample soils were taken from around the diesel stations and petroleum products storage tanks of Zanjan city, Iran. The samples were mixed and sieved using a 2 mm mesh screen. The samples contained 75% sand, 16% clay, 9% silt and 5.89% organic carbon. The soil sample was rinsed two times with distilled water and left on the filter paper to drain the excess water for 24 hours at room temperature (20 ± 2 ċ) and then dried in oven at 60 ċ for two hours.

### Washing assays

All experiments in this study were carried out using 100 g of soil in 500 mL Erlenmeyer after adding certain amounts of water, surfactants, chelating agent and adjusting the pH, they were placed in a shaker incubator (model JTSL40). TPH experiments were carried out in different operating variables including the different speed of agitation (100, 150, 200 and 250 rpm), contact times (10, 20, 30, 60, 90 and 120 min), concentrations of surfactants (2, 5, 10, 20, 30 and 60 g/kg) and pHs (2.5, 3, 5, 7, 7.5, 8.5 and 9). Then soil washing continued by adding 0.02 mole EDTA and NaCl simultaneously with the best results obtained from previous steps in order to investigate heavy metal removal. According to the findings of this study, EDTA had higher efficiency relative to NaCl in removing heavy metal, thus experiments carried out with EDTA alone in different concentrations of EDTA (0.01, 0.02, 0.5 and 0.1 mole) and pHs (2–8). After finishing the Washing assays, the suspension was filtered, dried and the TPH and Pb and Cd of samples were measured. The percent removal (%R) of pollutants was calculated using the following formula: %R = C_0_ – C/C_0 *_100. Where C_0_ and C were the concentrations of pollutants before and after washing, respectively.

### Analysis methods

**Figure 1:**
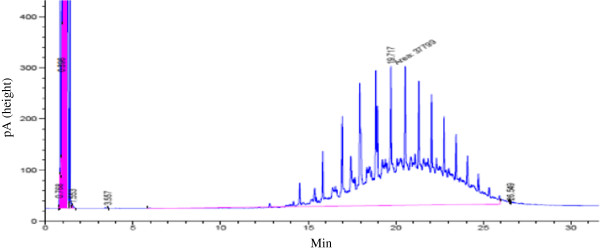
An example of graph obtained in this study.

The TPH concentration of samples was analyzed using GC-FID method. The GC unit (Agilent 7890A) was equipped with a flame ionization detector (FID) and a capillary column (25 m, long; 0.25 mm, ID; 0.25 μm film thickness). The TPH of the soils was extracted using liquid- solid extraction method by n-hexane according to TNRCC method [24]. The temperatures of injection port and detector were 250 and 325 ċ, respectively. oven temperature was kept constant at 45 ċ for 3 min, and then programmed with the rate of 12 ċ ⁄min, to reach 90 ċ with the hold time of 4 min and then to reach 275 ċ with the hold time of 12 min according to the test method SW-846 (3550B). In this study, two hydrocarbons of C_10_ and C_28_ were injected into gas chromatography set and by obtaining retention times 5.93 and 25.9, the surface under the curve between these two times was considered as concentration index. A graph obtained is shown in Figure 1. For analysis of heavy metals, after digestion of samples the concentrations of Pb and Cd were measured using atomic absorption spectrophotometer (Varian AA-240). In this study, 2 kinds of surfactants were used for washing of soils. The characteristics of these 2 surfactants are shown in Table 1. The chemicals used in the research were purchased from Merck Company. Non-ionic surfactants were purchased from Aldrich Company. In order to study the role of different operational parameters such as speed of agitation, contact time, etc. the washing assays were carried out in different stages. In each stage one of the parameters was variable and the others were constant. Selection of Tween 80 and Brij 35 and their concentrations in this study obtained from similar researches has been done by Mouton et al. and Peng et al. on PAHs removal from polluted soil [18,23].

**Table 1:** **Characteristic of applied surfactants in this research **[25]

	**Density (g/mL)**	**formula**	**CMC (mg/L)**
**Tween 80**	1	C_64_H_124_O_27_	16
**Brij 35**	1.05	CH_3_(CH_2_)_11_(OCH_2_CH_2_)n-OH	110

## Results and discussion

### Effect of agitation speed

**Figure 2:**
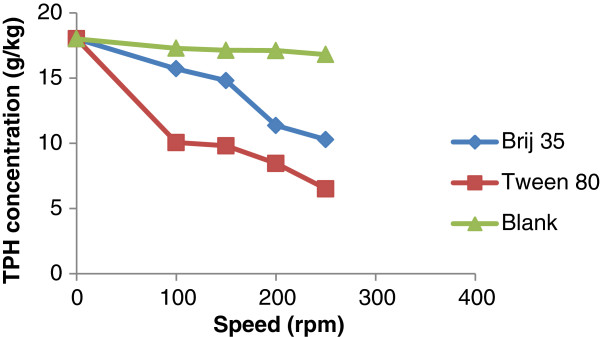
The effect of agitation speed on removal of TPH using pH slurry: 7, liquid to soil (w/w) 10:1, surfactant to soil: 5 g/kg, contact time: 30 min.

Agitation speed plays an important role in soil washing. In order to determine the optimum speed, washing were carried out in separate flasks in different speed and a flask with no surfactant use in control flask. The results are shown in Figure 2. TPH concentration has a tendency to decrease with increasing agitation speed to 250 rpm. Best TPH removal yields were obtained at 250 rpm for Tween 80 and Brij 35. The same results was observed by Peng et al. [18]. These researchers reported that the main reason is stronger collision between soil particles and increasing agitation speed which helps the stripping of the adsorbed or crusted contaminants.

### Effect of contact time

**Figure 3:**
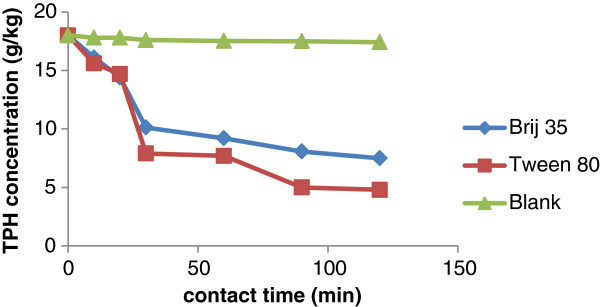
The effect of contact time on removal of TPH using pH slurry: 7; liquid to soil (w/w) 10:1; surfactant to soil: 5 g/kg; agitation speed: 250 rpm.

One of the important factors influencing the removal of pollutants from soils in washing process is contact time. In order to determine the optimum contact time in washing operations for TPH removal, a study was performed by washing of the soil in different contact times. The Effect of contact time results is shown in Figure 3. The TPH concentration has a tendency to decrease with increasing contact time to 90 min. The results showed that the distilled water cannot remove the TPH from soil (in control flask) the main reason is that the hydrocarbons in the TPH are no polar but water is a polar solution. The maximum efficiency of TPH removal in 90 min contact time was 71% in flask containing Tween 80 as washing surfactant and 54% in flask containing Brij 35 (Figure 3). The results of this test confirms researches of Moutsatsou et al. on the influence of time on the extraction of metals from soil from washing it with a 1 M HCl solution and 0.1 M EDTA solution [26].

### Effect of surfactant concentration

**Figure 4:**
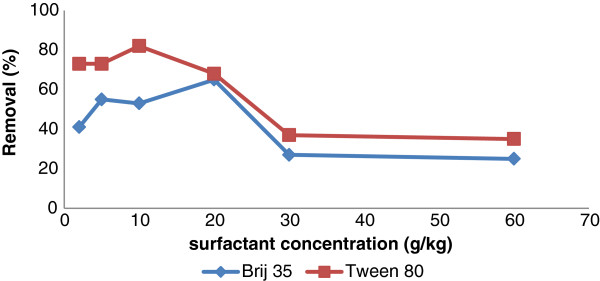
Removal efficienies of TPH by different concentration of surfactants using pH slurry: 7; liquid to soil (w/w) 10:1; agitation speed: 90 rpm; contact time: 90 min.

The soil washing operations were conducted using different concentration of surfactants. The results are shown in Figure 4. Best TPH removal yields (85% and 65%) were obtained at 10 g/kg Tween 80 and 20 g/kg Brij 35, respectively. During the washing assays it was revealed that the solubility of TPH from soil was decreased. Interaction between water, particles, metals and hydrophobic particles is the main reason for decreasing the TPH removal by high concentration of surfactants. Interfacial behavior of surfactants plays an important role in these observations. The first micelle formed in soil solution/system is introduced as CMC_eff_ (effective critical micelle concentration in soil/aqueous solution) by Zheng and Obbard [27]. In this study, the concentration at which the TPH solubility is maximal is named CMC_eff_. As shown in Table 1, the CMC of Tween80 and Brij35 are 16 and 110 mg/l, respectively. The solubility of TPH is high around the CMCeff. The CMC_eff_ values are estimated 1000 and 2000 mg/l for Tween80 and Brij35, respectively. These values are 62.5 and 18 times higher than CMC of Tween80 and Brij35, respectively. For concentration lower than the CMC_eff,_ the surfactants appear as soluble macromolecules in the medium and cannot interact with contaminants. The main interactions between surfactants and TPH take place around the CMC_eff_. While at higher than CMC_eff_, interactions with hydrophobic particles and mineral particles [18].

### Effect of pH on TPH removal

Effect of pH on TPH removal, at optimum agitation speed and contact time and concentration obtained from previous steps investigated. No TPH removal was observed and the results are not presented. The main reason is presence of different kinds of organic compounds involving aliphatic and aromatic hydrocarbons in petroleum fractions. Each of these compounds has specific chemical behavior at different pHs. Therefore it is impossible to attained specific pH to remove all of these compounds Mouton et al. investigated effects of pH on removal of PAHs in soil washing [18]. They observed that a decrease of the soil pH to 3 causes a considerable decrease of low (less than five aromatic rings) molecular weight PAH removal (45% to 16%). High molecular weight PAH (more than five aromatic rings) removal is maintained (45– 46%) in these conditions.

### Cd and Pb removal

The results of Cd and Pb removal in soil washing assays by different concentrations of non- ionic surfactants showed that the both non- ionic surfactants had weak capability for metals removal. This inability is due to lack of negative charge on the surface of surfactant molecules. Therefore NaCl and EDTA as chelating agents were added to the flasks. These agents have high efficiency of metal extraction from soil and they can form stable metal complexes in the soil-water mixture. The results are shown in Table 2. It is observed that NaCl has no significant effect on Cd and Pb removal, but the capability of EDTA on metals removal is high and acceptable. It should be mentioned that the concentration of Pb in the soil samples of this study was higher than the Cd concentration. The main reason is that there are many Pb mines in Zanjan. Tween 80 and Brij 35 found that EDTA is effective in Cd and Pb removal. EDTA addition increases the Cd and Pb removal with creating of EDTA complexes, such as ([Pb(EDTA)]^2−^,[Pb(HEDTA)]^−^, [Pb(H_2_EDTA)]) or ([Cd(EDTA)]^2−^,[Cd (HEDTA)]^−^, [Cd (H_2_EDTA)]) [28].

**Table 2:** Removal efficienies of Cd and Pb by 2 concentrations of Tween 80 and Brij 35 in the presence of EDTA and NaCl

	**Surfactant concentration**	**Pb Removal (%)**		**Cd Removal (%)**	
		**C**_**0**_ **= 350 mg/kg**		**C**_**0**_ **= 36 mg/kg**	
		**EDTA**	**NaCL**	**EDTA**	**NaCL**
**Tween 80**	10 g/kg	50	7	68	21
**Tween 80**	20 g/kg	62	6	73	30
**Brij 35**	10 g/kg	61	18	68	NO
**Brij 35**	20 g/kg	70	23	71	18

EDTA: 0.02 mol; NaCl: 0.02 mol; liquid to soil (w/w) 10:1; agitation speed = 90 rpm; pH slurry: 7 contact time: 90 min.

### Effect of pH on cadmium and lead removal

**Figure 5:**
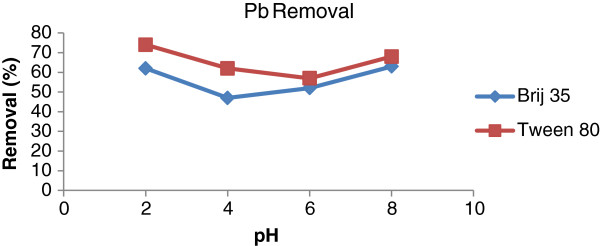
Removal efficienies of Pb by different pH using liquid to soil (w/w) 10:1; agitation speed: 90 rpm; contact time: 90 min; Brij 35 concentration: 20 g/kg; Tween 80 concentration: 10 g/kg.

**Figure 6:**
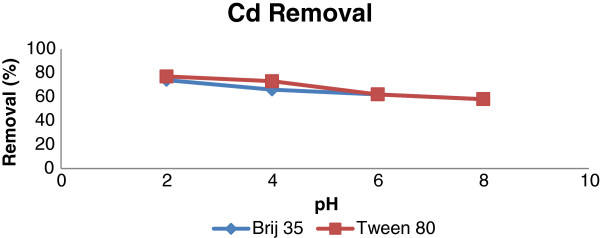
Removal efficienies of Cd by different pH using liquid to soil (w/w) 10:1; agitation speed: 90 rpm; contact time: 90 min; Brij 35 concentration: 20 g/kg; Tween 80 concentration: 10 g/kg.

In this stage, the soil washing experiments were carried out in different pHs. Because of the weak results of NaCl in removal of heavy metals, the soil washing tests were done with EDTA as chelating agent. The results which are presented in Figures 5 and 6 show that with increasing pH, the removal of Cd and Pb were decreased. The solubility of the metals in lower pH is high. Therefore, in this study the maximum removal of metals (metal complexes formation) occurred in pH = 2. In pHs higher than 8, the precipitations of Cd (OH) _2_ and Pb (OH) _2_ is formed. Lead can also be removed by precipitation as carbonate. The pH required in this case is between 7.5 and 8.5; therefore precipitation at pH higher than 8, as well as the surfactant effects plays a role in the removal of metals in the experiments, we also investigated net amount of surfactants on removal of lead and cadmium by Tween 80, the highest removal was about 23%. It was cleared that the normal pH of the slurry of the soil samples was about 6. The differences between the efficiencies of the metals removal in acidic pHs and normal pH of the slurry of the soil (6.8-7.2) were not significant, thus, the normal pH of the soil was considered as optimum for metals removal. On the other hand, the cost of decreasing pH should be considered.

### Removal of metals by different concentrations of EDTA

**Figure 7:**
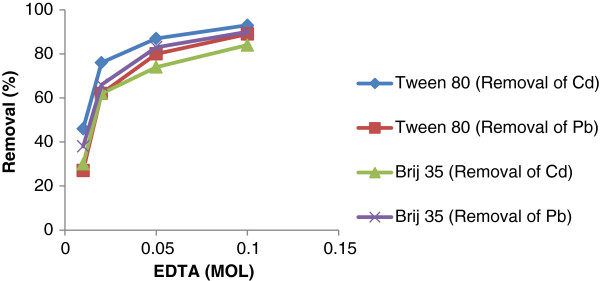
Removal efficienies of Cd and Pb by different concentrations of EDTA.

The soil washing experiments were conducted with concentration of 1, 20, 50 and 100 mmol EDTA in normal pH and pre optimized operational conditions. The results are shown in Figure 6. the removal efficiency of metals increased with increasing of EDTA concentration either in the case of Tween 80 and Brij 35. Results of Figure 7 agreed with Mahvi et al. [8]. who found the removal efficiency of Cd, Pb and Zn increased with increasing of concentration of EDTA from 0.005 – 0.1 M.

## Conclusion

Washing of soil by tween 80 is an effective and quick method and can be used for remediation of petroleum contaminated soil. It can be a good choice for remediation of both heavy metals and diesel contaminated sites. Although this method was tested for remediation of diesel contaminated soil, it also can be proposed for other soils contaminated with petroleum hydrocarbons. EDTA increases the solubility of heavy metals. Operating conditions which obtained in the present research should be tested on actual sites with organic and inorganic contaminants.

## Competing interests

The authors declare that they have no competing interests.

## Authors’ contributions

Authors contributed to the article as follows: MB was responsible for study design, sample collection, data analysis, summarization of results, interpretation of results, and manuscript preparation. MRM supervised the study. He was involved in study design and setting up gas chromatography and atomic absorption sets and participated in measuring heavy metals and TPHs. AA and MMF were advisors the study and participated in designing the field studies. They gave general support in carrying out the study. MM helped in preparation and editing manuscript. FR helped in preparation of samples, digestion of soil and data analysis. All authors read and approved the final manuscript.
