# Correction: Efficiency of non-ionic surfactants - EDTA for treating TPH and heavy metals from contaminated soil

**DOI:** 10.1186/2052-336X-12-47

**Published:** 2014-02-24

**Authors:** Mansour Baziar, Mohammad Reza Mehrasebi, Ali Assadi, Mehran Mohammadian Fazli, Mohammad Maroosi, Fooad Rahimi

**Affiliations:** 1 Department of Environmental Health Engineering, School of Public Health, Tehran University of Medical Science, Tehran, Iran; 2 Department of Environmental Health, Faculty of Health, Zanjan University of Medical Sciences, Zanjan, Iran; 3 Department of Health, Neyshabur University of Medical Sciences, Neyshabur, Iran

## 

Journal of Environmental Health Science and Engineering 2013, 11:41. doi:10.1186/2052-336X-11-41

## Correction

After publication of this article [1], we noticed that, Maroosi M and Rahimi F should remove from paper according to their own desires and at the request of project supervisor professor. Please see below, that is a corrected version of authors and their affiliation.

Authors

Mansour Baziar^1^*, Mohammad Reza Mehrasebi^2^, Mehran Mohammadian Fazli^2^, Ali Assadi^2^

*Corresponding author

^1^Department of Environmental Health Engineering, School of Public Health, Tehran University of Medical Science, Tehran, Iran

^2^Department of Environmental Health, Faculty of Health, Zanjan University of Medical Sciences, Zanjan, Iran
